# Organophosphorus Pesticides Management Strategies: Prohibition and Restriction Multi-Category Multi-Class Models, Environmental Transformation Risks, and Special Attention List

**DOI:** 10.3390/toxics13010016

**Published:** 2024-12-26

**Authors:** Yingwei Wang, Lu Wang, Yufei Li

**Affiliations:** 1 Colleges of Forestry, Northeast Forestry University, 26 Hexing Road, Harbin 150040, China; wyw102000@126.com; 2 Jilin Province Ecological Environmental Monitoring Centre, 813 Pudong Road, Changchun 130011, China; winniewanglu@163.com

**Keywords:** organophosphate pesticides, machine learning, environmental transformation products, toxicity characteristics, special attention list

## Abstract

Organophosphorus pesticides (OPs) have become one of the most widely used pesticides in Chinese agriculture; however, methods to identify potential restrictions on OPs molecules are lacking. Therefore, this study retrieved the OPs restriction list and constructed eight multi-class, multi-category machine learning models for OPs restrictions. Among these, the random forest (RF) model demonstrated excellent predictive performance, as it was successfully validated and applied. Potential environmental transformation products of OPs were obtained using EAWAG-BBD software, while toxicity indicators for the parent OPs and their transformation products were predicted with ADMETlab 3.0 software. This study found that unrestricted OPs, such as phorate, parathion, and chlorpyrifos, exhibited a high probability of toxicity. Additionally, the environmental transformation products of OPs posed similar comprehensive toxicity risks as the parent compounds. A special attention list for OPs was created based on the toxicity risks of unrestricted parent OPs and their transformation products, using standard deviation classification. Phorate and parathion were identified as OPs requiring special attention. This paper aims to provide an effective method for identifying the potential restriction levels of OPs and to propose an evaluation system that comprehensively considers the health risk, thereby supporting the improvement and optimization of management and usage strategies for OPs.

## 1. Introduction

As agriculture becomes increasingly industrialized, the usage of pesticides has steadily increased to protect crops from pests and diseases [1]. Organophosphorus pesticides (OPs), one of the most widely used pesticides in agriculture, have a higher acute toxicity than organochlorines but, fortunately, a shorter half-life [2,3]. However, the improper use of OPs has led to significant environmental residues, posing serious threats to ecosystems and human health [4]. Studies have shown that acute or chronic exposure to OPs can affect humans, animals, plants, and insects to varying degrees, causing respiratory, reproductive, and neurotoxic effects [5]. In humans and animals, OPs primarily bind to acetylcholinesterase (AChE) in the nervous system, reducing AChE activity and causing acetylcholine to accumulate in the synaptic cleft, thereby impairing nerve transmission [6]. Patients chronically exposed to OPs typically exhibit symptoms such as seizures, confusion, muscle pain, and shortness of breath [7]. Therefore, the proper use and management of OPs are essential to mitigating their negative impacts on ecosystems and human health.

Most OPs sprayed to eliminate pests can migrate from farmlands to aquatic habitats via surface runoff [8]. Recently, researchers have detected OPs in various environmental media, including surface water [9,10] and agricultural soil [11]. OPs not only affect target organisms but also pose significant health risks to various non-target organisms, such as earthworms, bees, fish, and birds [12]. The environmental transformation products of OPs are potential pollutants in ecosystems. However, most current research focuses on the toxicity and hazards of OPs themselves, with little attention given to their environmental transformation products. Studies have found that residual OPs in the environment can undergo degradation through processes such as hydrolysis, photolysis, and redox reactions. Their transformation products possess different physicochemical properties and may exhibit greater biotoxicity and environmental persistence than the parent OPs [13]. Recent studies show that 3,5,6-trichloropyridinol, the primary metabolite of chlorpyrifos, infiltrates the soil environment more readily than its parent compound, impacting soil microbial communities and exerting toxicological effects on earthworms [14]. Additionally, Wei et al. [15] identified OPs metabolites, including methyl parathion and methyl paraoxon, in Taihu Lake, with high environmental residue concentrations posing significant toxicological risks to aquatic organisms. Therefore, evaluating the toxicity of OPs and their transformation products is crucial for comprehensively understanding their environmental impact. Such assessments help more accurately gauge the potential threats OPs pose to ecosystems and human health, guiding more effective OPs management and usage strategies.

Due to its simple algorithmic programming and efficient computational capabilities, machine learning can more effectively discover patterns and trends in complex data [16]. By learning from a portion of the data, these methods can then be used to predict and evaluate the remaining data [17]. In recent years, machine learning classification models have been widely applied in environmental science and engineering. Liu et al. [18] utilized machine learning classification models, such as decision trees and random forests, to classify large-scale microplastics by their spectral data, enhancing the potential of machine learning to identify microplastics in real-world environments. Lombard et al. [19] employed boosted regression trees and random forest machine learning models to effectively predict human health risks under arsenic exposure. Currently, the Pesticide Management Regulations issued by the Ministry of Agriculture and Rural Affairs of the People’s Republic of China, along with the regulations from the U.S. Environmental Protection Agency (EPA) and the European Union (EU), classify OPs into three categories: banned, restricted, and unrestricted. Recent studies indicate that the primary OPs types found in groundwater and surface water in China were restricted and unrestricted pesticides, such as omethoate, dichlorvos, trichlorfon, and malathion. Crustaceans exhibit high sensitivity to these substances, with unrestricted OPs (dichlorvos) already causing significant adverse effects on non-target aquatic organisms like fish [20,21]. The above analysis indicates that, in addition to prohibited OPs, some widely used OPs molecules still pose considerable toxicity risks to aquatic organisms. There is an urgent need for a model that can effectively evaluate and predict the restriction, prohibition, and non-restriction levels of OPs molecules. Such a model can support OPs molecular screening, evaluation, and management strategies while minimizing large-scale resource consumption in in vitro and in vivo experiments. Furthermore, machine learning exhibits significant advantages in identifying, training, and predicting the potential environmental effects of emerging pollutants. Therefore, this study constructed a multi-class, multi-category model for OPs restriction and prohibition using machine learning algorithms to effectively identify the restriction levels of OPs molecules, providing important theoretical support for improving China’s OPs restriction and prohibition list.

To investigate the issues outlined above, this study primarily involved the following: (1) After retrieving OPs pesticide management lists published by the EPA, EU, and the Ministry of Agriculture and Rural Affairs of the People’s Republic of China, we constructed a multi-class machine learning model for the banned and restricted use of OPs using eight algorithms: random forest (RF), logistic regression (LR), support vector machine (SVM), complement naive Bayes (CNB), K-nearest neighbor (KNN), eXtreme gradient boosting (XGBoost), gradient boosting machines (GBM), and artificial neural network (ANN). It established a comprehensive model evaluation metric system to assess model effectiveness and validated and applied the multi-class machine learning model for OPs based on the organophosphorus pesticide management levels of the EPA and EU. (2) EAWAG-BBD software was utilized to identify 79 potential environmental transformation products of OP molecules, predicting the toxicity of both parent OPs and their transformation products to comprehensively evaluate the toxicity characteristics of OPs and their environmental derivatives. (3) Based on the comprehensive toxicity risks of unrestricted OPs and their transformation products, a special attention list for OPs was compiled. This study aimed to preliminarily construct a model capable of identifying and predicting the restriction, prohibition, and non-restriction (unregistered) levels of OPs pesticide molecules in the EPA, EU, and Chinese databases. This will accelerate the process of advancing OPs molecules into in vitro and in vivo experiments and their eventual registration while minimizing the potential adverse effects on non-target organisms during development and use. Furthermore, it provides robust technical support for the screening, evaluation, and management strategy development of novel OPs molecular alternatives. At the same time, based on a comprehensive assessment of the toxicity risks of OPs and their transformation products, we propose priority and special attention molecular lists. This aimed to provide data support for accurately evaluating the potential impacts of OPs on ecosystems and human health and to offer a theoretical basis for the comprehensive formulation of OPs management and usage strategies.

## 2. Materials and Methods

### 2.1. OPs Molecular Structure Source and Dataset Construction—Database Retrieval Method

This paper reviewed the “Pesticide Management Regulations” issued by the Ministry of Agriculture and Rural Affairs of the People’s Republic of China (http://www.moa.gov.cn/, accessed on 10 September 2024) and identified 17 banned OPs, 8 restricted OPs, and 54 unrestricted OPs (Table 1) for the dataset. Additionally, this study utilized the pesticide information website (https://www.pesticideinfo.org/, accessed on 10 September 2024) to retrieve data on 199 OPs molecules registered in the EPA and EU databases. The molecular structures of these OPs were sourced from the National Library of Medicine’s database (https://pubchem.ncbi.nlm.nih.gov/, accessed on 10 September 2024) using the PubChem module, and their 3D structures were obtained using the 3D Conformer module.

### 2.2. Acquisition and Dimensionality Reduction of OPs Molecule Descriptors—PaDEL-Descriptor and Pearson Correlation Coefficient Method

Molecular descriptors transform the structural information of chemical molecules into useful numerical data or standardized experimental results and are widely used in the field of computational chemistry [22]. The PaDEL-Descriptor 2.2.1 software was used to calculate 1444 descriptors for 79 OPs molecules in this study, including electronic, structural, physicochemical, and topological parameters, to characterize the structural variations among different OPs molecules. However, the PaDEL-Descriptor software failed to accurately recognize some OPs molecules, resulting in missing values for some molecular descriptors. Furthermore, high-dimensional datasets can lead to overfitting during model construction, slow computation speeds, and extensive memory usage, making it challenging to build reliable machine learning models [23]. Consequently, the Pearson correlation coefficient feature selection method was employed to handle missing values and eliminate redundant features from the 1444 OPs molecular descriptors in this study, resulting in a remaining 905 descriptors. Through Pearson correlation coefficient filtering, a final set of 78 molecular descriptors that characterize key structural features of OPs molecules was selected, which were then used as independent variables in subsequent studies.

### 2.3. Construction Method for a Multi-Class Machine Learning Model for Restricted and Unrestricted OPs Molecules—Machine Learning

This study retrieved data on the current regulatory status of 79 known OPs molecules in China as well as their registration statuses with the EPA and EU. The 79 OPs molecules were classified into banned, restricted, and unrestricted categories (model classification defined as y1); the EPA categories were primarily YES or NO (model classification defined as y2), and the EU categories were Yes, No, and Not listed (model classification defined as y3). Consequently, in this paper, the classifications of the 79 OPs were used in China, the EPA, and the EU as dependent variables, with the 78 key descriptors of OPs selected in Section 2.2 serving as independent variables, to construct a multi-class machine learning model for the regulated use of OPs molecules. This aimed to expedite the identification of potential classifications of OPs molecules, thereby accelerating the development of new OPs compounds. This study employed a variety of algorithms, including RF, XGBoost, CNB, GBM, KNN, LR, ANN, and SVM, to build a multi-class machine learning model for regulated use of OPs molecules. This study was conducted on the Windows operating system using the Python programming language and the Jupyter Notebook development tool to construct and predict machine learning models. The detailed algorithms can be found in Supplementary Information Text S1.

### 2.4. Evaluation Metrics for a Multi-Class Machine Learning Model for Restricted and Unrestricted OPs Molecules

To evaluate the predictive performance of the multi-class classification model for regulated use of OPs, a confusion matrix was utilized as a statistical classification technique to provide a clear assessment of the correct and incorrect predictions in this study [24]. The structure of the confusion matrix is illustrated in Table 2. The elements of the confusion matrix, including true positives (TP), false positives (FP), true negatives (TN), and false negatives (FN), were used to assess the accuracy of the predictions made by the multi-class classification model for regulated use of OPs.

In this study, eight multi-class classification models were evaluated for regulated use of OPs based on accuracy, recall, precision, and F1 score metrics to select the most precise model. The detailed formulas can be found in Supplementary Information Text S2.

### 2.5. Prediction of Toxicity Risk for OPs Molecules and Their Environmental Transformation Products—EAWAG-BBD and ADMETlab 3.0 Software

OPs could cause various toxic effects, including hepatic and neurotoxic effects, posing severe threats to human health and the ecological environment [25]. Similarly, the transformation products of OPs have demonstrated potential impacts on biodiversity in soil, water, and the atmosphere [26]. The EAWAG-BBD pathway prediction system can identify the functional groups of OPs molecules and predict the transformation products of OPs based on biotransformation rules [27]. Additionally, the ADMET evaluation module of ADMETlab 3.0 software can calculate potential human health and environmental risks of various organic compounds, including hERG blockers, hERG blockers (10 μm), DILI, AMES toxicity, rat oral acute toxicity, FDAMDD, skin sensitization, carcinogenicity, eye corrosion, eye irritation, respiratory toxicity, human hepatotoxicity, drug-induced nephrotoxicity, drug-induced neurotoxicity, ototoxicity, nematotoxicity, genotoxicity, RPMI-8226 immunotoxicity, A549 cytotoxicity, Hek293 cytotoxicity, BCF, IGC_50_, LC_50_DM, and LC_50_FM [28]. Therefore, in this study, the EAWAG-BBD pathway prediction system was used to obtain the environmental transformation products of 79 OPs molecules, and ADMETlab 3.0 software was applied to calculate the toxicity data for OPs and their transformation products, thereby assessing their toxicological risks.

### 2.6. Comprehensive Toxicity Assessment of OPs Molecules and Their Transformation Products—Coupling Entropy Weighting Method with TOPSIS

#### 2.6.1. Calculation of Toxicity Index Weights for OPs Molecules and Their Transformation Products—Entropy Weighting Method

The entropy weighting method utilizes information entropy to assign objective weights. It provides insights based on the significance of each index, offering reliable data and reducing biases caused by subjective factors such as human error [29]. Consequently, the entropy weighting method was applied to calculate the information entropy of various toxicity parameters of OPs molecules in this study. A lower entropy value indicates a higher weight and a greater potential impact on the ecological environment. Additionally, to standardize the variation and trends among OPs toxicity indicators such as BCF, IGC_50_, LC_50_DM, and LC_50_FM with other toxicity metrics, this study conducted positive normalization for BCF parameters and negative normalization for IGC_50_, LC_50_DM, and LC_50_FM parameters. The detailed calculation formulas can be found in Supplementary Information Text S3.

#### 2.6.2. Assessment of Comprehensive Toxicity Risk for OPs Molecules and Their Transformation Products—TOPSIS Method

To comprehensively and systematically analyze the toxicity risks of OPs and their transformation products, the entropy weighting method coupled with the TOPSIS method was employed for an integrated evaluation of their toxicity in this study. TOPSIS is a commonly used multi-criteria decision analysis method that ranks alternatives based on their closeness to an ideal solution, using a distance-based comprehensive evaluation approach [30]. The TOPSIS method was used, and the distances to the positive and negative ideal solutions were calculated, with a composite index reflecting how closely the evaluation criteria approached the optimal solution [31]. The detailed calculation formulas can be found in Supplementary Information Text S4.

### 2.7. Development of a Special Attention List for Unrestricted-Class OPs Molecules—Standard Deviation Classification Method

To further assess the comprehensive toxicity risks of unrestricted OPs and their transformation products and to propose a special attention list to maximize prevention of potential health and ecological risks from these substances, this study calculated the average values of 24 toxicity indicators for 54 types of unrestricted OPs molecules and their transformation products; averaged the toxicity indicator data across all transformation products of different OPs molecules; and used these average values to characterize the overall toxicity risks of OPs and their transformation products. A standard deviation classification method was employed to draft a special attention list for unrestricted OPs, offering theoretical guidance for refining China’s pesticide restriction list. The detailed calculation formulas can be found in Supplementary Information Text S5.

## 3. Results and Discussion

### 3.1. Analysis of Feature Variables in a Multi-Class Classification Model for Restricted and Unrestricted OPs Molecules

To construct feature variables for a multi-class classification model of regulated use of OPs molecules, the method described in Section 2.2 was used to obtain 78 key molecular descriptors of OPs in this study. Additionally, the RF feature selection method was applied to calculate the importance of these features (Table 3).

Broto–Moreau autocorrelation descriptors significantly influenced the multi-class classification model for regulated use of OPs, such as AATSC7i (average centered Broto–Moreau autocorrelation—lag 7/weighted by first ionization potential), ATSC4c (centered Broto–Moreau autocorrelation—lag 4/weighted by charges), and ATSC5s (centered Broto–Moreau autocorrelation—lag 5/weighted by I-state). These descriptors quantified internal structural characteristics of OPs molecules, detailing the average distances and connections between atoms, reflecting the three-dimensional structural features, and underscoring the importance of atomic topological autocorrelation in predicting OPs classification outcomes. Among them, the AATSC7i descriptor had the highest feature importance (1.0000), was related to the molecular ionization potential, and was positively correlated with the chlorination level of OPs [32], suggesting that OPs containing chlorine posed a higher health risk and should receive special attention. Furthermore, Wang et al. [33] used OPs molecular descriptors to develop and screen for highly functional and environmentally friendly OPs substitute molecules, with AATSC7i also serving as a key characteristic descriptor, consistent with the findings of this study. Geary autocorrelation descriptors, specifically the GATS series, characterized the chemical structure of OPs molecules by calculating autocorrelations of specific properties between atom pairs in OPs molecules, such as GATS3e (Geary autocorrelation—lag 3/weighted by Sanderson electronegativities) and GATS3c (Geary autocorrelation—lag 3/weighted by charges). Autocorrelation indices reflected the distribution of atomic properties along the topological structure, encoding both the molecular structure and numerical properties of atoms, such as atomic mass, volume, polarizability, and electronegativity, used as weighting properties [34]. The Barysz matrix, based on molecular topology and geometric information, is typically used to compute molecular topological properties, such as VE2_DzZ (average coefficient sum of the last eigenvector from the Barysz matrix, weighted by atomic number) and VE2_Dzm (average coefficient sum of the last eigenvector from the Barysz matrix, weighted by mass). The 78 molecular descriptors selected as feature variables were utilized to construct a multi-class classification model for regulated use of OPs molecules in this study, which could enhance the accuracy of the machine learning multi-class classification model for OPs molecules.

### 3.2. Evaluation of a Multi-Class Machine Learning Model for Restricted and Unrestricted OPs Molecules

To select the best-performing multi-class classification model for regulated use of OPs, this study constructed eight models based on 78 molecular descriptors selected using a Pearson correlation coefficient feature selector. The 79 OPs were split into training and test sets at a 7:3 ratio, and the predictive performance of the multi-class classification models for OPs molecules was assessed based on a set of evaluation metrics. Evaluation metrics from the confusion matrix, along with accuracy, precision, recall, and F1 score, were used to assess the predictive performance of the eight multi-class classification models for regulated OPs molecules. The models with superior predictive performance were then selected, with results shown in Table 4, and the range of hyperparameters and values is shown in Table S1.

Multi-class classification models for regulated OPs molecules constructed using RF, XGBoost, and CNB algorithms demonstrated good classification performance. Simulation results indicated that the RF-constructed multi-class classification model for OPs molecules yielded nearly ideal outcomes for y1 classification, with all metrics above 0.8. For y2 and y3, the precision and F1 score were 0.7656 and 0.8167, respectively, suggesting that the RF model (hereinafter referred to as the OP multi-class classification model) exceled in predicting y1. The XGBoost-constructed multi-class classification model performed well for the y2 classification, with all evaluation metrics exceeding 0.8. The performance metrics for y1 and y3 were lower than those for y2, indicating that XGBoost was particularly effective in classifying y2. Compared to multi-class classification models constructed with RF and XGBoost algorithms, the GBM algorithm’s model performed particularly well in classifying y3, with all evaluation metrics exceeding 0.9, indicating that the GBM model exceled in classifying y3. The RF algorithm’s multi-class classification model for OPs molecules showed excellent performance, with all evaluation metrics around 0.8. In comparison to the GBM algorithm, the XGBoost algorithm’s multi-class classification model for OPs molecules incorporated regularization, which helped reduce overfitting and simplified the decision trees generated by the model, thus reducing its complexity. Additionally, all performance metrics for the CNB algorithm’s multi-class classification model for regulated OPs exceeded 0.7, indicating the good adaptability of CNB for multi-class issues. During the construction process, CNB focused on the complement of each category to calculate the classification probability for each category of OPs molecules, thus performing better in managing class imbalances [35]. The ANN possessed strong nonlinear modeling capabilities and can address multiple tasks simultaneously [36]. The multi-class classification model constructed using the ANN algorithm showed excellent results for the y2 category, with all performance metrics exceeding 0.9. However, the training process for the ANN algorithm was complex and data-intensive, typically favored for binary classification tasks. It had certain limitations in multi-class tasks [37], which may have affected its overall performance in multi-class classification models for OPs molecules. KNN assigned labels to new samples based on the predominant label among the K-nearest samples. This method tended to favor the majority class when predicting unknown samples, leading to significant errors in the multi-class classification models for OPs molecules constructed using KNN [38]. Similar to LR, the SVM algorithm demonstrated exceptional performance in binary classification tasks. However, extending it to a multi-class classification model for regulated OPs required a “one-vs-one” strategy, which increased not only the computational complexity but also the complexity of the model [39]. Consequently, as indicated in Table 4, the multi-class classification models for OPs molecules constructed using GBM, KNN, LR, ANN, and SVM showed poor predictive performance for the three-category y1, with precision, recall, and F1 score all below 0.7.

In summary, the predictive performance of multi-class classification models for regulated OPs molecules should be evaluated comprehensively based on four key metrics: accuracy, precision, recall, and F1 score. A model was considered to have excellent classification performance if these metrics reached 0.7 or higher. Consequently, the multi-class classification models for OPs molecules built using the RF, XGBoost, and CNB algorithms met these criteria, demonstrating good predictive performance.

### 3.3. Validation and Application of a Multi-Class Machine Learning Model for Restricted and Unrestricted OPs Molecules

This study successfully constructed eight multi-class classification models for regulated OPs molecules, and based on four evaluation metrics (accuracy, precision, recall, and F1 score), all exceeding 0.7, we selected three models with outstanding predictive performance. Among them, the RF algorithm’s model for OPs achieved all four evaluation metrics at 0.8 for y1 classification. Consequently, this study used the RF-based multi-class classification model for OPs molecules as an example, validating its predictive performance against the OPs pesticide lists managed by the EPA and the EU.

In this study, the EPA and EU OPs management lists were retrieved, identifying 29 OPs molecules marked as “No” on these lists but not included on China’s restricted list of OPs (Table S2) to validate the multi-class classification model for regulated use of OPs molecules. In the EU OPs management list, the multi-class classification model for OPs molecules constructed using the RF algorithm demonstrated excellent predictive performance, with all four evaluation metrics above 0.6 (precision: 0.6897; accuracy: 1.000; recall: 0.6897; F1 score: 0.8163), indicating strong predictive capabilities. On the EPA OPs management list, the predictive performance of the model was less impressive, with only two of the four evaluation metrics exceeding 0.6 (precision: 0.5862; accuracy: 1.000; recall: 0.5862; F1 score: 0.7391), showing limited predictive capabilities. Further analysis indicated that due to the limited number of “Yes” samples in the EPA OPs management list, the multi-class classification models for regulated OPs molecules were undertrained, leading to biased predictions. However, the small difference between the probabilities of correct and incorrect predictions suggests that the RF-based model still had some capacity for error correction. Additionally, using the Pearson correlation coefficient method to select molecular descriptors may impact the prediction results, mainly because the chosen descriptors might not fully represent the structural characteristics of OPs molecules. To further apply the multi-class classification model for regulated OPs molecules, in this study, 170 OPs molecules from the EU OPs management list were elected that were unregistered and not listed on China’s restricted list. Predictions were made using the RF-based multi-class machine learning model (Table 5) to assess the potential classification levels of these 170 OPs molecules within China’s OPs management list. The predictions indicated that out of the 170 unregistered EU OPs molecules, 75, 6, and 89 were classified as prohibited, restricted, and unrestricted, respectively. This suggests that these OPs molecules may pose potential risks and management needs. This application demonstrates the potential of machine learning models in chemical risk assessment, providing data support for future policy formulation.

### 3.4. Prediction and Analysis of Comprehensive Toxicity Risk and Characteristics of Unrestricted-Class OPs Molecules

#### 3.4.1. Analysis of Variability Among Toxicity Indices of Unrestricted-Class OPs Molecules

In this study, the toxicity tool of the ADMET evaluation module was employed to calculate toxicity indices such as hERG blockers, DILI, BCF, and AMES toxicity for 54 unrestricted-class OPs molecules in China (Table S3). Analysis of 24 toxicity indices for these molecules revealed significant variations in the evaluation results. Compared to the other 23 toxicity indices, genotoxicity was the most pronounced in these molecules, with 92.6% having a high probability of genotoxicity occurrence (>99.0%) and 38.0% showing a probability of 100.0% for genotoxicity manifestation. Joshi et al. reported that malathion and several other OPs exert negative effects on the male reproductive system [40]. Furthermore, the 54 unrestricted OPs molecules in China showed pronounced hepatotoxicity and respiratory toxicity, with the proportion of molecules having a toxicity probability over 95.0%, reaching 88.8% and 74.1%, respectively. Studies indicated that prolonged exposure to these OPs molecules can lead to severe liver fibrosis and liver dysfunction, with steatohepatitis being a typical manifestation of liver damage [41]. Furthermore, OPs can irreversibly bind to the AChE enzyme, leading to respiratory diseases in poisoned individuals, with severe cases potentially resulting in death due to respiratory distress [6].

#### 3.4.2. Analysis of Comprehensive Toxicity Characteristics of Different Unrestricted-Class OPs Molecules

To analyze the comprehensive toxicity of different unrestricted-class OPs molecules in China, the entropy weighting method coupled with the TOPSIS method was employed to conduct a comprehensive evaluation of 24 toxicity indices in this study. Initially, the entropy weighting method was used to further analyze the contribution of toxicity indices of unrestricted-class OPs molecules in China, with ototoxicity having the highest weight (18.017%), indicating that ototoxicity significantly contributed to the overall toxicity of OPs molecules. However, the probability of ototoxicity occurring in the 54 OPs molecules was low; therefore, it was likely that the high weighting was due to significant variations in the incidence of ototoxicity among different OPs molecules. The average values of 24 toxicity indices were calculated for 54 unrestricted-class OPs molecules in China, and a comprehensive assessment of their overall toxicity was conducted using the TOPSIS method, based on the assigned weights to these indices (Table S3). Although tetrachlorvinphos was considered an efficient and low-toxicity OP, it can inhibit normal human AChE activity [42]. Analysis of Table S3 revealed that tetrachlorvinphos exhibits the highest overall toxicity, with probabilities of genotoxicity, respiratory toxicity, and liver damage at 100.0%, 99.3%, and 99.9%, respectively. It also showed probabilities greater than 99.9% for nephrotoxicity, dermatoxicity, and route of administration (ROA) toxicity. Additionally, other highly toxic OP molecules include chlorfenvinphos and propaphos, with chlorfenvinphos showing probabilities of 99.9%, 99.1%, and 99.7% for genotoxicity, respiratory toxicity, and liver damage, respectively, and with propaphos showing probabilities of 99.9%, 99.6%, and 99.9%. Recent studies indicated that chlorfenvinphos can cause severe neurotoxicity and liver damage [43], while propaphos effectively inhibited AChE activity, leading to neurological disorders [44]. Therefore, although tetrachlorvinphos, chlorfenvinphos, and propaphos are unrestricted in China, their potential genotoxicity, respiratory toxicity, and hepatotoxicity still require significant attention. Prolonged use may have considerable adverse effects on both the environment and human health, necessitating urgent attention.

### 3.5. Prediction and Analysis of Comprehensive Toxicity Characteristics of OPs Molecular Environmental Transformation Products

#### 3.5.1. Prediction and Analysis of Variability in Toxicity Among OPs Molecular Environmental Transformation Products

Environmental transformation products of OPs molecules exhibited high probabilities of eye irritation, respiratory toxicity, and dermatoxicity. Studies showed that OPs molecules decompose in the natural environment through hydrolysis, photodegradation, and bacterial degradation, among other conditions. However, research on the toxicity of transformation products was relatively limited, and in certain cases, these products may exhibit greater toxicity risks than the parent OPs molecules. Initially, using the method described in Section 2.5, this study predicted and identified 332 potential environmental transformation products of 79 OPs molecules. Subsequently, the Toxicity tool of the ADMET evaluation module was utilized to predict 24 toxicity indices in this study, including hERG blockers, DILI, BCF, and AMES toxicity, for 332 transformation products of OPs molecules (Table S4). Analysis of 24 toxicity indices for 332 transformation products of OPs molecules revealed significant variability among different toxicity indices. Compared to the other 23 toxicity indices, eye irritation was the most pronounced in the 332 environmental transformation products of OPs molecules, with 80.1% showing a probability of eye irritation greater than 95.0%. Existing research has shown that local exposure of malathion to the rat eye can cause temporary ocular irritation [45], findings that bear similarities to the results of this study. Additionally, the environmental transformation products of 332 OPs molecules exhibited high rates of respiratory, dermatoxic, and genotoxic effects, with proportions exceeding a 95.0% occurrence probability at 55.4%, 68.1%, and 73.2%, respectively. Notably, some environmental transformation products of non-restricted OPs molecules still exhibit high toxicity. Among these, genotoxicity was the most pronounced, with an occurrence probability greater than 95.0% in 84.1% of the products, significantly higher than those of prohibited- and restricted-class OPs molecules. Consequently, the use of some unrestricted-class OPs should be a matter of significant attention.

#### 3.5.2. Analysis of Comprehensive Toxicity Characteristics of Different OPs Molecular Environmental Transformation Products

The environmental transformation products of OPs molecules exhibited similar comprehensive toxicity risks as their parent OPs molecules. To analyze the comprehensive toxicity of 332 different environmental transformation products of OPs molecules, the method described in Section 2.6 was employed to assess 24 toxicity indices of these products in this study. Initially, the entropy weighting method was utilized to further analyze the variability in toxicity indices of unrestricted-class OPs molecular transformation products in China. The results indicated that ototoxicity had the highest weight (13.889%), suggesting that ototoxicity significantly contributed to the overall toxicity of OPs transformation products. Subsequently, the average values of 24 toxicity indices for 332 OPs molecular transformation products were calculated, and their overall toxicity was assessed using the TOPSIS method based on their toxicity weights. Finally, the comprehensive toxicity of the 332 OPs transformation products was adjusted by multiplying the average values of the molecular toxicity indices by their TOPSIS scores (Table S4). Analysis revealed that the transformation product of tetrachlorvinphos, 2-chloro-1-(2,4,5-trichlorophenyl) ethenone, exhibited the highest comprehensive toxicity, with probabilities of causing dermatoxicity, respiratory toxicity, and eye irritation at 99.9%, 99.9%, and 99.6% respectively. This toxicity was second only to tetrachlorvinphos itself, underscoring the need for careful monitoring during its use. Additionally, dichlofenthion, an unrestricted-class OP molecule, had a transformation product, 2,4-dichlorophenol, which exhibited significant comprehensive toxicity, notably in eye irritation at 99.4%. Therefore, the use of dichlofenthion should also be closely monitored.

To further analyze the comprehensive toxicity of OPs molecular environmental transformation products, the average comprehensive toxicity of 54 such OPs products was calculated in this study. Analysis revealed that the six environmental transformation products of fenamiphos exhibited the highest average comprehensive toxicity. Research indicated that due to fenamiphos’s high solubility, it can persist in both soil and aquatic environments, demonstrating high toxicity to non-target organisms, including fish, earthworms, and birds [46]. Although fenamiphos was classified as a prohibited OP molecule, its transformation products still exhibit high toxicity. Among restricted-class OPs molecules, the four transformation products of acephate exhibited the highest average comprehensive toxicity, with particularly notable genotoxicity. Related research has also found that acephate can lead to diseases such as diabetes, anemia, and Alzheimer’s disease [47]. Among unrestricted-class OPs molecules, the two environmental transformation products of phorate also showed high average comprehensive toxicity, particularly as discussed in Section 3.4.2. Additionally, while phosalone, an unrestricted-class OP molecule, exhibited lower comprehensive toxicity, its six environmental transformation products showed higher comprehensive toxicity. Among these, the toxicity of diethyl dithiophosphate was particularly significant. Bo et al. [48] found that diethyl dithiophosphate was associated with impaired glucose homeostasis and an increased risk of type 2 diabetes in adolescents. Therefore, both restricted- (such as acephate) and unrestricted- (such as phorate and fenthion) class OPs, along with their transformation products, still posed significant toxicity risks with the potential to severely harm human health and the environment, and they should therefore be closely monitored.

### 3.6. The Establishment of a Watch List Focusing on the Comprehensive Toxic Risks of OPs and Their Transformation Products

To further mitigate the risks that OPs molecules posed to human health and the environment, this paper, based on a comprehensive assessment of the toxicity risks of OPs and their transformation products, proposes a special attention list for unrestricted-class OPs molecules. The method described in Section 2.7 was employed to determine the comprehensive toxicity of unrestricted-class OPs molecules and their transformation products in this study. Among these, the standard deviation δ of the comprehensive toxicity for the 54 unrestricted-class OPs molecules and their transformation products was 0.0459. This study set 1.85δ as a scoring interval, categorizing unrestricted-class OPs molecules within the ranges of (−3.7*δ*, −1.85*δ*], (−1.85*δ*, 0], (0, 1.85*δ*], and (1.85*δ*, 3.7*δ*] into four levels of attention: special, high, medium, and low, respectively (Table 6). Analysis has determined that tetrachlorvinphos and chlorfenvinphos should be given special attention as OPs molecules, a finding consistent with the results analyzed in Section 3.4 and Section 3.5 of this study. Recent studies, including one conducted by Wang et al. [49], identified 77 organophosphate compounds in soil and aquatic environments, with tetrachlorvinphos presenting a substantial threat to aquatic ecosystems. Additionally, research has found that tetrachlorvinphos is carcinogenic, inducing tumors in mouse hepatocytes and renal tubules [50]. Vegetables may contain residues of chlorfenvinphos, which, when ingested, inhibit AChE activity and pose serious health threats [51]. The analysis presented above confirms the validity of the special attention list for unrestricted-class OP compounds developed in this study.

## 4. Conclusions

This study, based on the EPA and EU databases and the OPs management classifications published by the Ministry of Agriculture and Rural Affairs of the People’s Republic of China, employed eight machine learning algorithms—RF, LR, SVM, CNB, KNN, XGBoost, GBM, and ANN—to construct a multi-class, multi-category prediction model for OPs, both restricted and banned. The results indicate that the RF, XGBoost, and CNB algorithms demonstrated outstanding classification performance. The constructed models achieved or exceeded the expected standards in external validation and application evaluation metrics, effectively evaluating the possible banned, restricted, or unrestricted (unregistered) levels of OPs molecules. Furthermore, based on the comprehensive toxicity assessment results of OPs, this study proposes priority toxicity indicators for OPs and their transformation products, including genotoxicity, hepatotoxicity, ocular irritation, respiratory toxicity, and dermal toxicity. Additionally, a list of OPs requiring special attention was identified, such as tetrachlorvinphos and chlorfenvinphos. The findings of this study provide critical data support for the efficient management and optimized usage strategies of OPs while highlighting the potential threats posed by OPs and their transformation products to ecosystems and human health. Future research should further expand the applicability of the model and incorporate more environmental factors to improve the accuracy and practicality of predictions, thereby promoting the development of OPs management strategies toward greater scientific precision and refinement.

## Figures and Tables

**Table 1:** China’s management list of banned and restricted OPs pesticides.

Level	Name	Level	Name
Banned	Methamidophos	Unrestricted	Pyraclofos
	Parathion		Propaphos
	Methyl parathion		Prothiofos
	Monocrotophos		Dichlofenthion
	Phosphoric acid		Pyridiphenthion
	Fenamiphos		Fosthietan
	Fonofos		Tebupirimfos
	Phosfolan-methyl		Chlorfenvinphos
	Cadusafos		Diazinon
	Coumaphos		Isoxathion
	Sulfotep		Azinphos-ethyl
	Terbufos		Dibrom
	Methidathion		Famphur
	Phorate		Phosalone
	Isofenphos-methyl		Heptenophos
	Isocarbophos		Acethion
	Ethoprophos		Azamethiphos
Restricted	Omethoate		Chlorpyrifos-methyl
	Systox		Pirimiphos-methyl
	Posfolan-methyl		Thiometon
	Isazophos		Phosphocarb
	Acephate		Sulprofos
	Dimethoate		Chlormephos
	Chlorpyrifos		Chlorethoxyfos
	Triazophos		Isophenphos
Unrestricted	Fenthion		Mecarbam
	Profenofos		Naftalofos
	Dichlorvos		Tetrachlorvinphos
	Fosthiazate		Cyanophos
	Phenthoate		Temephos
	Dipterex		Mevinphos
	Phoxim		Fensulfothion
	Quinalphos		Mecarbam
	Fenitrothion		Phosemet
	Malathion		Oxydemeton-methyl
	Propetamphos		Disulfoton
	Dicrotophos		Ethion
	Azinphos-methyl		Thionazin
	EPN		Kitazine
	Pyrazophos		

**Table 2:** Confusion Matrix output structure.

Confusion Matrix		True		
		P	N	M
Estimate	P	TP	FP_1_	FP_2_
	N	FN_1_	TN	FN_2_
	M	FM_1_	FM_2_	TM

Note: P represents banned, N represents restricted, and M represents unrestricted.

**Table 3:** Ranking of key feature descriptors by importance in a multi-class machine learning model for 78 restricted and unrestricted OPs molecules.

Feature	Importance	Relative Importance	Feature	Importance	Relative Importance	Feature	Importance	Relative Importance
AATSC7i	0.0403	1.0000	VR2_Dzp	0.0138	0.3295	ATSC4v	0.0089	0.2077
VE2_DzZ	0.0302	0.7437	mindssC	0.0135	0.3227	BCUTc-1l	0.0088	0.2031
ATSC4c	0.0293	0.7231	ATSC8p	0.0135	0.3225	ATSC1e	0.0087	0.2005
VE2_Dzp	0.0292	0.7198	AATS5s	0.0133	0.3167	minHBa	0.0087	0.2004
GATS3e	0.0282	0.6945	ALogp2	0.0131	0.3136	ATSC6e	0.0086	0.1990
GATS3c	0.0273	0.6704	ATSC2e	0.0128	0.3053	AATS7i	0.0086	0.1982
GATS1m	0.0247	0.6056	AATSC8i	0.0128	0.3051	TopoPSA	0.0084	0.1941
VE3_Dzm	0.0237	0.5803	VE2_Dze	0.0125	0.2974	VC-4	0.0082	0.1888
ASP-5	0.0224	0.5467	VE2_Dt	0.0123	0.2929	AATS7s	0.0080	0.1828
GATS4c	0.0216	0.5275	ATSC4s	0.0121	0.2878	MATS2s	0.0078	0.1778
ATSC2c	0.0208	0.5077	AATSC3m	0.0114	0.2707	WTPT-5	0.0070	0.1596
JGI7	0.0206	0.5024	topoShape	0.0111	0.2629	VR2_DzZ	0.0065	0.1446
ASP-7	0.0198	0.4810	JGI8	0.0110	0.2597	AATSC7c	0.0059	0.1298
GATS2m	0.0197	0.4788	JGI3	0.0110	0.2585	JGI2	0.0056	0.1240
MDEC-13	0.0192	0.4669	AATS8s	0.0107	0.2513	nAtomLC	0.0049	0.1045
GATS2s	0.0191	0.4651	AATSC0i	0.0105	0.2467	SC-5	0.0048	0.1039
ATSC5s	0.0191	0.4642	GATS3s	0.0103	0.2413	apol	0.0047	0.0997
AATS1i	0.0189	0.4580	MATS2m	0.0101	0.2365	minsssCH	0.0034	0.0664
ATSC6p	0.0172	0.4159	VE2_Dzv	0.0099	0.2320	JGI5	0.0030	0.0585
JGI6	0.0172	0.4157	SpMin8_Bhi	0.0099	0.2308	minsCl	0.0022	0.0371
SpMin6_Bhi	0.0170	0.4111	GATS7i	0.0098	0.2290	SC-4	0.0021	0.0354
ATSC8e	0.0168	0.4061	MDEC-11	0.0098	0.2285	nAtomLAC	0.0021	0.0340
MLFER_BO	0.0167	0.4043	AATSC6m	0.0097	0.2262	ETA_dAlpha_B	0.0015	0.0187
AATSC7s	0.0165	0.3998	ATSC5p	0.0096	0.2254	MDEC-24	0.0012	0.0109
MDEO-22	0.0158	0.3802	VE2_Dzs	0.0094	0.2201	minHCsatu	0.0011	0.0106
SpMin7_Bhi	0.0147	0.3523	ASP-6	0.0090	0.2102	minwHBd	0.0007	0.0000

**Table 4:** Calculation of evaluation metrics for eight multi-class machine learning models for restricted and unrestricted OPs molecules.

Model	Classification	Accuracy	Precision	Recall	F1 Score
RF	y1	0.8750	0.8496	0.8750	0.8377
	y2	0.8750	0.7656	0.8750	0.8167
	y3	0.8750	0.7656	0.8750	0.8167
XGBoost	y1	0.7917	0.7976	0.7917	0.7873
	y2	0.8750	0.9148	0.8750	0.8944
	y3	0.8333	0.7822	0.8333	0.8040
CNB	y1	0.7083	0.7326	0.7083	0.7193
	y2	0.7500	0.9078	0.7500	0.8214
	y3	0.7500	0.7639	0.7500	0.7500
GBM	y1	0.7500	0.6902	0.7500	0.6688
	y2	0.8750	0.7656	0.8750	0.8167
	y3	0.9167	0.9167	0.9167	0.9167
KNN	y1	0.7500	0.7319	0.7500	0.6715
	y2	0.7917	0.9104	0.7917	0.8469
	y3	0.7500	0.7875	0.7500	0.7683
LR	y1	0.7083	0.6932	0.7083	0.6308
	y2	0.8333	0.7609	0.8333	0.7955
	y3	0.8750	0.8370	0.8750	0.8556
ANN	y1	0.7500	0.7734	0.7500	0.7598
	y2	0.9583	0.9184	0.9583	0.9379
	y3	0.7500	0.6818	0.7500	0.7143
SVM	y1	0.7917	0.7536	0.7917	0.7252
	y2	0.8750	0.8370	0.8750	0.8556
	y3	0.8333	0.6944	0.8333	0.7576

**Table 5:** Application and prediction of a multi-class classification model for restricted and unrestricted OPs molecules developed using the RF algorithm.

Name	Prediction	Name	Prediction
(E)-Mevinphos	Banned	Fospirate	Banned
(Z)-Mevinphos	Banned	Gardona (trans-isomer)	Unrestricted
2,5-dichloro-alpha-(chloromethylene)benzyl diethyl phosphate	Banned	Hexylthiofos	Banned
2,5-dichloro-alpha-(dichloromethylene)benzyl diethyl phosphate	Unrestricted	Imicyafos	Banned
Acetoxon	Banned	Inezin	Unrestricted
Akton	Unrestricted	Iprobenfos	Banned
alpha,alpha-Dithiol bis(O,O-dimethyl phosphorodithioate)toluene	Unrestricted	IPSP	Banned
Amidithion	Unrestricted	Isazophos-methyl	Unrestricted
Aminofenitrothion	Unrestricted	Isochlorthion	Unrestricted
Amiprofos-methyl	Unrestricted	isofenphos-methyl	Banned
Amiton oxalate	Unrestricted	Isomalathion (metabolite of malathion)	Unrestricted
Amiton	Unrestricted	Isophos	Banned
Aspon	Banned	Isothioate	Banned
Athidathion	Unrestricted	Kitazine	Banned
Azethion	Unrestricted	Leptophos	Unrestricted
Azinphos-methyl oxygen analog	Banned	Lythidathion	Banned
Azothoate	Unrestricted	Malaoxon	Unrestricted
Bensulide Oxon (degradate of bensulide)	Banned	Malathion dicarboxylic acid	Restricted
Bensulide	Banned	Mecarphon	Banned
Bomyl	Banned	Menazon	Banned
Buminafos	Unrestricted	Merpofos	Unrestricted
Butamifos	Unrestricted	Methyl paraoxon	Unrestricted
Butathiofos	Unrestricted	Methyl phenkapton	Banned
Butonate	Unrestricted	Methylisocyanothion	Unrestricted
Calvinphos	Unrestricted	Monocrotophos (isomer unspecified)	Unrestricted
Carbophenothion-methyl	Banned	Morphothion	Restricted
Chlorfenvinphos alpha	Banned	Naled	Unrestricted
Chlorfenvinphos-methyl	Unrestricted	Naphthalophos	Unrestricted
Chlorphoxim	Banned	O,O,S-Trimethyl phosphorodithiate (as impurity)	Unrestricted
Chlorprazophos	Unrestricted	O,O-diethyl O-naphthaloximido phosphorothioate	Unrestricted
Chlorpyifos oxygen analog	Banned	O,O-diethyl phosphoro chloridothionate	Banned
Chlorthion	Unrestricted	O,O-Diethyl S-(4,6-dimethyl-2-pyrimidinyl) phosphorodithioate	Banned
Chlorthiophos I	Unrestricted	O,O-diethyl-O-phenyl phosphorothioate	Unrestricted
Chlorthiophos II	Unrestricted	O,O-dimethyl S-(2-(ethylsulfinyl)-1-methylethyl) phosphorothioate	Banned
Chlorthiophos III	Unrestricted	O-(2-chloro-4-nitrophenyl) O-isopropyl ethylphosphonothioate	Unrestricted
cis-Azodrin	Unrestricted	O-(4-Nitrophenyl) O-phenyl methylphosphonothioate	Unrestricted
cis-Methocrotophos	Banned	O-ethyl O-(4-(methylthio)phenyl) methylphosphonothioate	Banned
Coumaphos oxon (metabolite of coumaphos)	Unrestricted	O-Ethyl O-methyl S-propylphosphorothioate (metabolite of ethoprop)	Banned
Coumithioate	Banned	O-Ethyl S-(4-methylphenyl) ethylphosphonodithioate	Banned
Crotoxyphos	Banned	Oxydemeton methyl sulfone	Unrestricted
Crufomate	Restricted	Paraoxon	Banned
Cyanofenphos	Unrestricted	Phenkapton	Banned
Cyanthoate	Banned	Phenthoate acid (as impurity)	Unrestricted
Cythioate	Unrestricted	Phorate oxygen analog sulfone	Unrestricted
Demephion-O	Restricted	Phorate oxygen analog	Banned
Demephion-S	Restricted	Phorate sulfone	Unrestricted
Demeton-O-methyl	Banned	Phoratoxon sulfoxide	Banned
Demeton-O	Unrestricted	Phosacetim	Unrestricted
Demeton-S sulfone	Banned	Phosfolan-methyl	Banned
Demeton	Banned	Phosmet	Banned
Dequest 2010 phosphonate	Unrestricted	Phosmetoxon	Banned
Desbromoleptophos	Unrestricted	Phosnichlor	Unrestricted
Desmethyl fenitrothion (metabolite of fenitrothion)	Banned	phosphocarb	Unrestricted
Desmethyl malathion (metabolite of malathion)	Banned	Phosphoric acid, 2-(1,1-dimethylethyl)-5-pyrimidinyl ethyl 1-methylethyl ester	Unrestricted
Phosphorodithioic acid, O-methyl ester, S-ester with 2-mercapto-N-methylacetamide, S-potassium salt	Banned	Phosphorothioic acid, S-((6-chloro-2-oxo-3(2H)-benzoxazolyl) methyl) O,O-diethyl ester	Banned
Desmethylfenitrothion	Unrestricted	Phostebupirim	Unrestricted
Diamidafos	Banned	Phoxim-methyl	Banned
Diazoxon	Banned	Piperophos	Unrestricted
Dimethyl chlorothiophosphate	Banned	Pirimiphos ethyl, O-analog	Unrestricted
Dioxabenzofos	Unrestricted	Pirimiphos-methyl	Banned
Dioxathion	Unrestricted	Primidophos	Unrestricted
Diphenprofos	Unrestricted	Propoxon	Banned
Disulfoton sulfone oxygen analog	Unrestricted	Prothidathion	Banned
Disulfoton sulfone	Banned	Prothion	Banned
Disulfoton sulfoxide	Banned	Quadrafos	Unrestricted
DMCP	Banned	Quinalphos-methyl	Unrestricted
Endothion	Unrestricted	Quinothion	Unrestricted
EPBP	Unrestricted	Quintiofos	Unrestricted
Ethephon	Unrestricted	S,S,S-tributyl phosphorotrithioate	Banned
Ethion dioxon	Unrestricted	S-(1,1-Dimethylethyl) O-ethyl ethylphosphonothioate	Banned
Ethion, O-analog	Banned	S-(4-(1,1-dimethylethyl)phenyl) O-ethyl ethylphosphonodithioate	Unrestricted
Ethyl (2-mercaptoethyl) carbamate, S-ester of O,O-dimethyl phosphorodithioate	Restricted	S-Methyl fenitrothion	Unrestricted
Ethyl hydrogen 1-propylphosphonate	Unrestricted	Sarin (Nonpesticidal chemical)	Banned
Famphur, O-analog	Unrestricted	Sophamide	Unrestricted
Famphur	Unrestricted	Sulprofos oxon	Banned
Fenamiphos sulfone	Banned	Temephos sulfoxide	Unrestricted
Fenamiphos sulfoxide	Banned	TEPP	Unrestricted
Fenitrothion oxygen analog	Unrestricted	Terbufos oxon sulfone	Unrestricted
Fenthion oxon sulfone	Unrestricted	Terbufos sulfone	Unrestricted
Fenthion oxon sulfoxide	Banned	Terbufos sulfoxide	Banned
Fenthion oxon	Banned	Terbufos-O analog	Unrestricted
Fenthion sulfone	Unrestricted	Tetrachlorvinphos, Z-isomer	Unrestricted
Fenthion sulfoxide	Unrestricted	Thion	Banned
Fonofos oxon	Banned	Thionazin, O-analog	Banned
Fosmethilan	Banned	Tolclofos-methyl	Unrestricted

**Table 6:** The establishment of a watch list focusing on the comprehensive toxic risks of OPs and their transformation products.

Level	OPs	Comprehensive Toxicity	Level	OPs	Comprehensive Toxicity
Special Focus	Tetrachlorvinphos	0.5886	General Focus	Azinphos-ethyl	0.4191
	Chlorfenvinphos	0.5080		Fosthietan	0.4174
Focus	Propaphos	0.5037		Dicrotophos	0.4167
	Naftalofos	0.4994		Pirimiphos-methyl	0.4158
	Phoxim	0.4840		Thiometon	0.4156
	Dichlorvos	0.4798		Oxydemeton-methyl	0.4151
	Temephos	0.4673		Pyraclofos	0.4146
	Propetamphos	0.4655		Diazinon	0.4138
	Dichlofenthion	0.4543		Pyridiphenthion	0.4076
	Chlorpyrifos-methyl	0.4538		Azinphos-methyl	0.4059
	Sulprofos	0.4511		Isophenphos	0.4059
	Cyanophos	0.4506		Profenofos	0.4022
	Mevinphos	0.4494		Thionazin	0.4021
	EPN	0.4481		Fensulfothion	0.4015
	Dibrom	0.4361		Kitazine	0.4008
	Prothiofos	0.4358		Mecarbam	0.4003
	Isoxathion	0.4349		Phosemet	0.3878
	Fenthion	0.4330		Fosthiazate	0.3871
	Azamethiphos	0.4322		Acethion	0.3843
	Disulfoton	0.4308		Tebupirimfos	0.3823
	Fenitrothion	0.4303		Phosphocarb	0.3785
	Quinalphos	0.4286		Chlorethoxyfos	0.3693
	Phosalone	0.4267		Vamidothion	0.3599
	Ethion	0.4242		Pyrazophos	0.3502
	Heptenophos	0.4240		Dipterex	0.3479
	Chlormephos	0.4232	Secondary Focus	Phenthoate	0.3321
General Focus	Famphur	0.4227		Malathion	0.3097

## Data Availability

The original contributions presented in this study are included in the article/supplementary material. Further inquiries can be directed to the corresponding author.

## Supplementary Materials

The following supporting information can be downloaded at: https://www.mdpi.com/article/10.3390/toxics13010016/s1.
