# Supplementary material for: Organophosphorus Pesticides Management Strategies: Prohibition and Restriction Multi-Category Multi-Class Models, Environmental Transformation Risks, and Special Attention List
Source: Toxics. 2024 Dec 26;13(1):16. doi: 10.3390/toxics13010016 (PMC11768814; doi:10.3390/toxics13010016)
Supplement: Supplementary file 1 [file toxics-13-00016-s001.zip › toxics-3308695-supplementary.pdf]

Supplementary Information

Text S1 Construction method for a multi-class machine learning model for restricted and unrestricted OPs molecules — Machine learning

The RF algorithm was used to construct a multi-class classification model for regulated use of OPs molecules. RF was a supervised ensemble learning model used for classification, which processes the predictions from multiple independent weak learners. The decision tree model typically yields relatively good predictions and was a representative example of the Bagging ensemble algorithm. It consisted of a "forest" of many decision trees, and the final prediction was derived by aggregating the predictions from all trees. Key hyperparameters included the number of trees and the depth of each tree [1]. Therefore, the RF algorithm was employed to build a multi-class classification model for the regulated use of OPs molecules in this study.

The XGBoost algorithm was used to construct a multi-class classification model for the regulated use of OPs molecules. XGBoost was a gradient boosting-based ensemble algorithm that incorporates regularization terms. It used decision trees as base units and leverages the capabilities of weak learners to achieve robust performance [2]. The XGBoost algorithm was employed to build a multi-class classification model for the regulated use of OPs molecules in this study. The objective function of XGBoost, *L(θ)*, was calculated as follows [3]:

$$L(\theta) = \sum_{m} l(y_m, \widehat{y_m}) + \sum_{k} \Omega(f_k) \quad (1)$$

In the formula, m represented the number of OPs, i.e., m = 1, 2, ..., 79; k denoted the number of base tree models in the XGBoost prediction model, a hyperparameter; y was the classification level of OPs; and $\hat{y}$ was the predicted outcome of the OPs classification model by the algorithm.

The CNB algorithm was used to construct a multi-class classification model for the regulated use of OPs molecules. CNB was an enhancement of the Naive Bayes algorithm that reduces the influence of correlation among all features through mathematical methods, addressing the issue of sample imbalance [4]. Fasaee et al. [5] utilized the naive bayes algorithm to establish a classification model effectively predicting lead content in drinking water. Therefore, the CNB algorithm was employed to build a multi-class classification model for the regulated use of OPs molecules in this study. The formula for calculating the feature weights $\hat{\theta}_{i,y\neq c}$ that influence the classification levels of OPs was as follows:

$$\hat{\theta}_{i,y\neq c} = \frac{\alpha_i + \sum_{y_m \neq c} x_{im}}{\alpha_i n + \sum_{i,y\neq c} \sum_{i=1}^{n} x_{im}} \tag{2}$$

In the formula, m represented the number of OPs, i.e., m = 1, 2, ..., 79; x_{im} was the molecular descriptor under OPs-related feature i; α was the smoothing coefficient for CNB.

Based on the calculated feature weights $\hat{\theta}_{i,y\neq c}$, the formula for the CNB prediction function $p(y \neq c|X)$ was as follows:

$$p(y \neq c|X) = arg\ min_c \sum_i x_i \omega_{ci} \tag{3}$$

In the formula, ω_{ci} represented the logarithm of the feature weights influencing the classification level. By calculating the prediction function, one could determine the classification level corresponding to the minimum complement probability.

The GBM algorithm was used to construct a multi-class classification model for the regulated use of OPs molecules. The GBM algorithm was a powerful ensemble learning technique, similar to the RF algorithm. GBM sequentially constructs multiple weak learners to improve prediction accuracy, culminating in an aggregated prediction from a strong learner [6]. The GBM algorithm was employed to build a multi-class classification model for the regulated use of OPs molecules in this study. The computational formula was as follows:

$$f_m(x) = \sum_{m=1}^{M} \gamma_m h_m(x) \tag{4}$$

In the formula, m represented the number of OPs, that was, m = 1, 2, ..., 79; γ_m was the learning rate for OPs, which was also used for training a new weak learner in the GBM algorithm.

The KNN algorithm was employed to construct a multi-class classification model for the regulated use of OPs molecules. The KNN algorithm was a predictive statistical classification method that classifies objects in feature space based on the nearest training samples. It calculated the distance between samples using the Euclidean distance and sequentially optimizes the value of parameter k to obtain the optimal classification model [7]. Li et al. [8] used the KNN algorithm to establish a predictive classification model that effectively predicts the carcinogenicity of chemicals in rats. The KNN algorithm was employed to construct a multi-class classification model for the regulated use of OPs molecules in thin study.

The LR algorithm was employed to construct a multi-class classification model for the regulated use of OPs molecules. LR was a typical binary classification algorithm. Zhao et al. [9]

used the LR model to predict contaminants in crops grown in contaminated soils, effectively predicting cadmium contamination in rice. The loss function $J(\theta)$ for LR binary classification was as follows:

$$J(\theta) = -\sum_{i=1}^{m}\left[(y_i) * log\left(y_\theta(x_i)\right) + (1 - y_i) * \log\left(1 - y_\theta(x_i)\right)\right] \quad (5)$$

In the formula, θ represented the feature coefficients of the LR OPs classification prediction model; m denoted the number of OPs, i.e., m = 1, 2, ..., 79; y_i was the classification level of OPs; $y_\theta(x_i)$ was the OPs classification level calculated based on the feature coefficients θ; x_i were the molecular descriptors influencing OPs classification. This paper constructed a multi-class classification model for 79 regulated OPs molecules. Consequently, the softmax regression was incorporated to modify the loss function $J(\theta)$ in this study, adapting it for the multi-class classification tasks of OPs. The loss function for the LR multi-class classification, following the introduction of the softmax function, was as follows:

$$J(\theta) = -\sum_{i=1}^{m}\sum_{j=1}^{k} 1\{y_{(i)} = j\} * \log\left(\frac{e^{\omega_j * x_{(i)} + b_j}}{\sum_{l=1}^{k} e^{\omega_j * x_{(i)} + b_j}}\right) \quad (6)$$

In the formula, k was the classification level of OPs; $1\{\cdot\}$ was an indicator function that equals 1 when the predicted outcome from the LR OPs model matches the actual classification level (i.e., the model's judgment was correct), and 0 otherwise. ω_j was the weight corresponding to the j-th classification level of OPs, and b_j was the bias term for the j-th classification level. The probability of the j-th classification level for x_i was given by the softmax function: $\frac{e^{\omega_j * x_{(i)} + b_j}}{\sum_{l=1}^{k} e^{\omega_j * x_{(i)} + b_j}}$

The ANN algorithm was employed to construct a multi-class classification model for the regulated use of OPs molecules. The ANN algorithm was a computational model that simulates the structure and function of biological neural networks, inspired by the human brain. It consisted of numerous interconnected artificial neurons and includes input, hidden, and output layers [10]. The ANN algorithm was utilized to build a multi-class classification model for regulated use of OPs molecules in this study. The input layer consisted of key molecular descriptors of OPs, the hidden layers involved the computation of feature weights of these descriptors through activation functions, and the output layer provided the prediction results of the OPs classification model.

The SVM algorithm was employed to construct a multi-class classification model for the

regulated use of OPs molecules. The SVM algorithm was a particularly powerful and flexible supervised machine learning model. It classified by finding an optimal hyperplane in the feature space that maximizes the margin, capable of handling situations where the dimensionality of the data far exceeds the number of samples. Tan et al. [11] also effectively identified fecal contamination sources in geographically unrelated samples using the SVM algorithm. Consequently, the SVM algorithm was employed to construct a multi-class classification model for the regulated use of OPs molecules in this study, designing an SVM for any two classes of OPs samples.

Text S2 Evaluation metrics for a multi-class machine learning model for restricted and unrestricted OPs molecules

Accuracy was the most straightforward and intuitive metric in classification models, directly reflecting the effectiveness of the model's classification [12]. The calculation formula was as follows:

$$Accuracy = \frac{TP + TN + TM}{TP + FP_{1,2} + TN + FN_{1,2} + TM + FM_{1,2}} \quad (7)$$

Recall measured the proportion of correctly predicted samples out of the total actual samples, indicating the classification model's ability to predict minority classes [13]. The calculation formula was as follows:

$$Recall_P = \frac{TP}{TP + FN_1 + FM_1} \quad (8)$$

Precision represented the proportion of actual positives among those predicted as positive [14]. The calculation formula was as follows:

$$Precision_P = \frac{TP}{TP + FP_{1,2}} \quad (9)$$

The F1 score was the harmonic mean of precision and recall, balancing the precision and recall of the classification model [15]. The calculation formula was as follows:

$$F1\ Score_P = \frac{2 * precision_P * recall_P}{precision_P + recall_P} \quad (10)$$

Text S3 Calculation of toxicity index weights for OPs molecules and their transformation products—Entropy weighting method

$$z_{ij} = \frac{max(x_{ij}) - x_{ij}}{max(x_i) - min(x_i)} \quad (11)$$

$$z_{ij} = \frac{x_{ij} - min(x_{ij})}{max(x_i) - min(x_i)} \quad (12)$$

$$P_{ij} = \frac{z_{ij}}{\sum_i^n z_{ij}} \quad (13)$$

$$E_j=-\frac{1}{ln\,n}\sum_{i=1}^{n}p_{ji}\,ln\,p_{ij} \tag{14}$$

$$\omega_j=\frac{1-E_j}{\sum_{j=1}^{m}(1-E_j)} \tag{15}$$

$$D_j=1-E_j \tag{16}$$

$$B_i=\sum_{j=1}^{m}(\omega_j\times z_{ij}) \tag{17}$$

In the formula, P_{ij} represented the proportional value of the toxicity indicator for OPs and their transformation products; E_j was the information entropy value for OPs and their transformation products; D_j was the information utility value of the toxicity indicator for OPs and their transformation products; ω_j was the weight assigned to the toxicity indicator of OPs and their transformation products; B_i was the composite toxicity score of the toxicity indicator for OPs and their transformation products.

Text S4 Assessment of comprehensive toxicity risk for OPs molecules and their transformation products—TOPSIS method

$$D_i^+ = \sqrt{\sum_{j=1}^{m}\omega_j\left(Z_j^+ - z_{ij}\right)^2} \tag{18}$$

$$D_i^- = \sqrt{\sum_{j=1}^{m}\omega_j\left(Z_j^- - z_{ij}\right)^2} \tag{19}$$

$$C_i = \frac{D_i^-}{D_i^+ + D_i^-} \tag{20}$$

In the formula, D_i^+ represented the distance to the positive ideal solution; D_i^- represented the distance to the negative ideal solution. Z_j^+ was the maximum value of the toxicity parameter i for OPs and their transformation products, representing the positive ideal solution; Z_j^- was the minimum value of the toxicity parameter i, representing the negative ideal solution. ω_j was the weight assigned to the toxicity parameter of OPs and their transformation products. C_i was the composite toxicity score for OPs and their transformation products. A higher C_i value indicated higher overall toxicity.

Text S5 Development of a special attention list for unrestricted class OPs molecules—standard deviation classification method

$$\bar{x}=\frac{1}{n}\sum_{i=1}^{n}x_i \tag{21}$$

$$d_i=x_i-\bar{x} \tag{22}$$

$$\delta=\sqrt{\frac{1}{n}\sum_{i=1}^{n}d_i^2} \tag{23}$$

In the formula, $\bar{x}$ represented the average comprehensive toxicity value for unrestricted OPs and their transformation products; x_i refered to the i-th OPs molecule (i = 1, 2, ..., 54); n was the number of OPs molecules (n=54). d_i was the difference between the comprehensive toxicity data of the i-th OPs and their average value. S^2 was the variance of the comprehensive toxicity for 54 types of unrestricted OPs and their transformation products; δ was the standard deviation of the comprehensive toxicity for unrestricted OPs and their transformation products.

Reference

1. He, K.; Li, W.; Zhang, Y.; Sun, G.; McNulty, S.G.; Flanagan, N.E.; Richardson, C.J. Identifying driving hydrogeomorphic factors of coastal wetland downgrading using random forest classification models. *Sci. Total Environ.* **2023**, *894,* 164995. https://doi.org/10.1016/j.scitotenv.2023.164995
2. Li, L.; Qiao, J.; Yu, G.; Wang, L.; Li, H.Y.; Liao, C.; Zhu, Z. Interpretable tree-based ensemble model for predicting beach water quality. *Water Res.* **2022**, *211,* 118078. https://doi.org/10.1016/j.watres.2022.118078
3. Chen, Tianqi, and Carlos Guestrin. "Xgboost: A scalable tree boosting system." *Proceedings of the 22nd acm sigkdd international conference on knowledge discovery and data mining*. **2016**. https://doi.org/10.1145/2939672.2939785
4. Matsuda, H.; Shimoda, H.; Morikawa, T.; et al. Phytoestrogens from the roots of *Polygonum cuspidatum* (Polygonaceae): structure-requirement of hydroxyanthraquinones for estrogenic activity. *Bioorg. Med. Chem. Lett.* **2001**, *11*(14), 1839-1842. https://doi.org/10.1016/s0960-894x(01)00318-3
5. Fasaee, M.A.K.; Berglund, E.; Pieper, K.J.; Ling, E.; Benham, B.; Edwards, M. Developing a framework for classifying water lead levels at private drinking water systems: a Bayesian Belief Network approach. *Water Res.* **2021**, *189,* 116641. https://doi.org/10.1016/j.watres.2020.116641
6. Hsiung, S.Y.; Deng, S.X.; Li, J.; Huang, S.Y.; Liaw, C.K.; Huang, S.Y.; Hsieh, Y.S. Machine learning-based monosaccharide profiling for tissue-specific classification of *Wolfiporia extensa* samples. *Carbohydr. Polym.* 2023, *322*, 121338. https://doi.org/10.1016/j.carbpol.2023.121338
7. Saini, I.; Singh, D.; Khosla, A. QRS detection using K-nearest neighbor algorithm (KNN) and

evaluation on standard ECG databases. *J. Adv. Res.* **2013**, *4*(4), 331-344. https://doi.org/10.1016/j.jare.2012.05.007

8. Li, X.; Du, Z.; Wang, J.; et al. In silico estimation of chemical carcinogenicity with binary and ternary classification methods. *Mol. Inform.* **2015**, *34*(4), 228-235. https://doi.org/10.1002/minf.201400127
9. Zhao, C.; Yang, J.; Shi, H.; Chen, T. Transforming approach for assessing the performance and applicability of rice arsenic contamination forecasting models based on regression and probability methods. *J. Hazard. Mater.* **2022**, *424*, 127375. https://doi.org/10.1016/j.jhazmat.2021.127375
10. Hsiung, S.Y.; Deng, S.X.; Li, J.; Huang, S.Y.; Liaw, C.K.; Huang, S.Y.; Hsieh, Y.S. Machine learning-based monosaccharide profiling for tissue-specific classification of *Wolfiporia extensa* samples. *Carbohydr. Polym.* **2023**, *322*, 121338. https://doi.org/10.1016/j.carbpol.2023.121338
11. Tan, Q.; Li, W.; Chen, X. Identification of the source of fecal contamination for geographically unassociated samples with a statistical classification model based on support vector machine. *J. Hazard. Mater.* **2021**, *407*, 124821. https://doi.org/10.1016/j.jhazmat.2020.124821
12. Cui, Q.; Zhu, J.; Shu, J.; et al. Comprehensive evaluation of electric power prediction models based on DS evidence theory combined with multiple accuracy indicators. *J. Mod. Power Syst. Clean Energy* **2021**, *10*(3), 597-605. https://doi.org/10.35833/mpce.2020.000470
13. Mfateneza, E.; Rutayisire, P.C.; Biracyaza, E.; et al. Application of machine learning methods for predicting infant mortality in Rwanda: analysis of Rwanda demographic health survey 2014-15 dataset. *BMC Pregnancy Childbirth* **2022**, *22*(1), 388. https://doi.org/10.1186/s12884-022-04699-8
14. Moscatelli, M.; Parlapiano, F.; Narizzano, S.; et al. Corporate default forecasting with machine learning. *Expert Syst. Appl.* **2020**, *161*, 113567. https://doi.org/10.1016/j.eswa.2020.113567
15. DeVries, Z.; Locke, E.; Hoda, M.; et al. Using a national surgical database to predict complications following posterior lumbar surgery and comparing the area under the curve and F1-score for the assessment of prognostic capability. *Spine J.* **2021**, *21*(7), 1135-1142. https://doi.org/10.1016/j.spinee.2021.02.007
